# Examining the Role of Race in End-of-Life Care in the Intensive Care Unit: A Single-Center Observational Study

**DOI:** 10.1089/pmr.2023.0037

**Published:** 2023-09-11

**Authors:** Shahla Siddiqui, Diana Bouhassira, Lauren Kelly, Margaret Hayes, Austin Herbst, Sarah Ohnigian, Luke Hedrick, Kimberly Ona Ayala, Daniel S. Talmor, Jennifer P. Stevens

**Affiliations:** ^1^ Department of Anesthesia, Critical Care, and Pain Medicine, Beth Israel Deaconess Medical Center, Harvard Medical School, Boston, Massachusetts, USA.; ^2^ Department of Medicine, Beth Israel Deaconess Medical Center, Harvard Medical School, Boston, Massachusetts, USA.; ^3^ Department of Pulmonary Critical Care, Beth Israel Deaconess Medical Center, Harvard Medical School, Boston, Massachusetts, USA.

**Keywords:** bias, disparity, end of life, ethnicity, race

## Abstract

**Background:**

Prior studies have shown variation in the intensity of end-of-life care in intensive care units (ICUs) among patients of different races.

**Objective:**

We sought to identify variation in the levels of care at the end of life in the ICU and to assess for any association with race and ethnicity.

**Design:**

An observational, retrospective cohort study.

**Settings:**

A tertiary care center in Boston, MA.

**Participants:**

All critically ill patients admitted to medical and surgical ICUs between June 2019 and December 2020.

**Exposure:**

Self-identified race and ethnicity.

**Main Outcome and Measure:**

The primary outcome was death. Secondary outcomes included “code status,” markers of intensity of care, consultation by the Palliative care service, and consultation by the Ethics service.

**Results:**

A total of 9083 ICU patient encounters were analyzed. One thousand two hundred fifty-nine patients (14%) died in the ICU; the mean age of patients was 64 years (standard deviation 16.8), and 44% of patients were women. A large number of decedents (22.7%) did not have their race identified. These patients had a high rate of interventions at death. Code status varied by race, with more White patients designated as “Comfort Measures Only” (CMO) (74%) whereas more Black patients were designated as “Do Not Resuscitate/Do Not Intubate (DNR/DNI) and DNR/ok to intubate” (12.1% and 15.7%) at the end of life; after adjustment for age and severity of illness, there were no statistical differences by race for the use of the CMO code status. Use of dialysis at the end of life varied by self-identified race. Specifically, Black and Unknown patients were more likely to receive renal replacement therapy, even after adjustment for age and severity of illness (24% and 20%, *p* = 0.003).

**Conclusions:**

Our data describe a gap in identification of race and ethnicity, as well as differences at the end of life in the ICU, especially with respect to code status and certain markers of intensity.

## Key Points

**Question:** Does self-identified race and ethnicity correlate with differences in the intensity of end-of-life care provided to patients in an intensive care unit (ICU)?

**Findings:** Among the 1259 patients who died in critical care units in a single tertiary care center, we found significant differences across racial groups documented in the electronic health record in code status at death, length of stay, use of dialysis, and use of palliative care services. We found that more than 20% of decedents had an “Unknown” race and ethnicity.

**Meaning:** Significant differences exist within end-of-life care in the ICU among individuals with different races. Similar to reports in literature, a fifth of our cohort had missing race data and these patients were among the sickest. Further study is required to evaluate the etiology of these differences.

## Introduction

The Institute of Medicine describes a “good death” as one that is “free from avoidable distress and suffering for the patient, family, and caregivers, in general accord with the patient's and family's wishes, and reasonably consistent with clinical, cultural, and ethical standards.”^1^ Achieving this goal can be difficult for the large percentage of adult patients who die in the intensive care unit (ICU).^2^

Life-prolonging therapies are widely and readily available in the ICU environment but may not always be beneficial if they extend life in ways that are not associated with increased quality of life or in ways that conflict with a patient's preferences.^3^ Previous authors have identified disparities among racial and ethnic groups in code status at the end of life.^4^ Similarly, a recent systematic review has revealed the presence of structural inequalities in the ICU contributing to racial disparities in care.^5^

One possible explanation may be that individuals identifying with different racial and ethnic groups differ in how they wish to achieve a good death.^6^ Historically, ethical dilemmas in the ICU commonly arise in end-of-life care, and often these are triggered by tensions created by gaps in cultural understanding, expectations, and communication.^7^

Literature has pointed toward a greater gap when patients and families belong to a racial minority.^8,9^ In addition, prior studies using electronic health records (EHRs) suggest that 25%–57% of patients are missing race or ethnicity documentation.^10^ Ethnic categories provided in most U.S. databases are derived from the categories provided by the Office of Management and Budget (OMB) and include Hispanic/Latino or non-Hispanic ethnicity, whereas race categories generally include White, Asian (depicting of Chinese origin from Asia), Black and “Other” (which is a wide and generalized category including all who do not fit the above races).^11^

Given what has been stated earlier, we sought to use data from a retrospective ICU database to describe any differences in end-of-life care among critically ill patients of a diverse racial background at one tertiary care center in Boston. This study aims at adding to the existing literature that explores how patients of different racial backgrounds die in the ICU by specifically examining on a more granular level the clinical interventions that contribute to the intensity of medical care at the end of life, such as intubation, vasopressor use, tube feeding, and dialysis, as well as markers of communication such as Palliative care and Ethics consults.

Our hypothesis, based on previous literature, was that the racial background of patients dying in the ICU may correlate with the intensity of medical care provided at the time of death. We aim at generating further hypotheses from the results of this study.

## Methods

### Study population

We conducted a retrospective cohort study of all non-cardiac surgery ICU admissions among patients 18 years and older between July 2019 and December 2020 in a single tertiary care center in Boston, MA. This timeframe included patients admitted with COVID-19. This study was approved by the Beth Israel Deaconess Medical Center Institutional Review Board on 8.2.2021 (2021P000073), and all procedures were followed in accordance with the ethical standards of the responsible committee on human experimentation (institutional or regional) and with the Helsinki Declaration of 1975.

### Outcomes

Within our cohort, we identified the subset of patients who died during hospitalization (in the ICU or after care in the ICU). Restricting the cohort to these patients, we further identified code status and timing of the physician code status order placement based on our provider order entry system. Code status was divided into three designations: Do Not Resuscitate (DNR)/Ok to Intubate, DNR/Do Not Intubate (DNI), and Comfort Measures Only (CMO). We identified the last code status entered before death occurred in the ICU.

In addition, for patients who died during their admission to the ICU, we extracted clinical parameters that we considered markers of intensity of care. These included: intubation and the use of vasopressors at the time of death were identified as EHR signals that were persistent at the time of death. Ongoing renal replacement therapy, ongoing nutrition, Palliative care, and Ethics consults were extracted from the provider order entry system.

### Covariables

Patient demographics included age and gender at admission. Race and ethnicity were extracted from the hospital registration tables. Patient comorbidities were extracted using International Codes of Diseases (ICD) 10 codes based on the Charlson Comorbidities index or ICD 10 codes and diagnosis related group (DRG) for calculation of the Elixhauser comorbidity index.^12^

Severity of illness was measured with the sequential organ failure assessment (SOFA) score, collapsed into the maximum value in the first 24 hours of ICU admission.^13^ We further identified whether the patient was admitted to a surgical or medical ICU, as these ICUs are managed by different departments within our institution.

### Statistical analysis

Continuous data were summarized as mean (± standard deviation [SD]) or median (interquartile range [IQR]) and categorical variables as frequency (%). Normality was assessed with a Shapiro–Wilk test. For comparisons of continuous data between two groups (decedents vs. patients discharged alive and pre–COVID-19 vs. post–COVID-19), data were analyzed using a *t* test or Wilcoxon rank-sum test, as appropriate.

For comparisons of continuous data between more than two groups (i.e., across race categories), data were analyzed using one-way analysis of variance or Kruskal–Wallis test, as appropriate. Categorical data were assessed using a Chi-square or Fisher exact test, as appropriate. Included within the “Unknown race” category were those that declined to answer or could not answer due to clinical status, and “Other” included those that did not consider themselves represented by the categories provided.

Missing data elements such as race and ethnicity that were “missing not at random” were included as a separate category. Statistical analyses were performed using SAS software, version 9.4. Copyright © 2016 SAS Institute Inc. Multivariable logistic regression was also conducted using variables such as race and intensity of care at death adjusted for age and severity of illness, such as SOFA score.

We also conducted subgroup analysis to evaluate whether end-of-life care changed as a result of the COVID-19 pandemic, given the limited family presence during the COVID-19 pandemic due to infection control policies of our institution. This research followed the Strengthening the Reporting of Observational Studies in Epidemiology reporting guidelines for cohort studies. Two-sided *p*-values of <0.05 were considered statistically significant.

## Results

### Population description

**FIG. 1.**
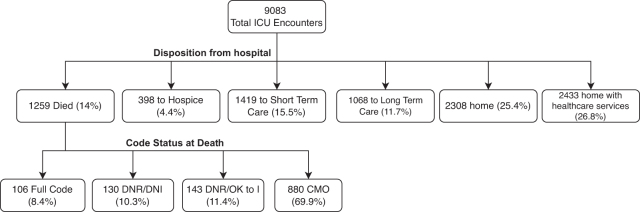
Discharge disposition and code status at death. CMO, Comfort Measures Only; DNI, Do Not Intubate; DNR, Do Not Resuscitate; ICU, intensive care unit.

A total of 9083 ICU patient encounters were included in this analysis. We included any patient admitted to a medical or surgical ICU during a hospital admission between July 2019 and December 2020 (Fig. 1). The majority of the patients were White (64%), followed by Black (14%), and “Unknown” race (13%). Overall, 4.8% were marked as “other” race (those whose race did not fit the categories provided, perhaps such as of the Indian subcontinent or Middle Eastern origin).

The mean age of the entire cohort was 64.4 (16.8 SD) years, and 44% were women. Of the entire cohort, 25% of the patients were discharged home with no assistance, 27% went home with health care services assistance, 14% died, and 4.4% were discharged to hospice (Supplementary Table S1a, b). The overall median hospital length of stay (LOS) for the cohort was 8 days (IQR 4, 15), and the median ICU LOS was 2.7 days (IQR 1, 6). The median SOFA score in the first 24 hours was 2 (IQR 1, 4).

Table 1 describes the demographic differences between those who died (decedents) (1259 or 13.9%) and those patients who were alive at discharge (7824 or 86.1%). Unsurprisingly, patients who died were older (69.4 (15.7 SD) years versus 62.6 (17.3 SD) years) (*p* < 0.0001) and had a comparatively higher SOFA score at 24 hours and a longer ICU LOS (*p* < 0.0001).

**Table 1.** Description of Cohort Divided By Survival and Death

Variable	All patients (***n*** = 9083), ***n*** (%)	Decedents (***n*** = 1259), ***n*** (%)	Alive at discharge (***n*** = 7824), ***n*** (%)	***p***
Age (years), mean (SD)	64.4 (16.8)	69.4 (15.7)	62.6 (17.3)	<0.0001
Gender	5092 (56.1)	714 (56.7)	4378 (56.0)	0.62
Male				
Ethnicity				<0.0001
Non-Hispanic	7404 (81.5)	934 (74.2)	6470 (82.7)	
Unobtainable	1088 (12)	263 (20.9)	825 (10.5)	
Hispanic/Latino	591 (6.5)	62 (4.9)	529 (6.8)	
Race				<0.0001
American Indian/Alaska	56 (0.6)	9 (0.7)	47 (0.6)	
Native and Native Hawaiian/Other Pacific				
Islander	331 (3.6)	39 (3.1)	292 (3.7)	
Asian	1293 (14.2)	166 (13.2)	1127 (14.4)	
Black	438 (4.8)	42 (3.3)	396 (5.1)	
Other	1182 (13.0)	286 (22.7)	896 (11.5)	
Unknown	5783 (63.7)	717 (57.0)	5066 (64.8)	
White				
Code status				<0.0001
Full code	6878 (75.7)	112 (8.6)	10,695 (89.8)	
DNR/DNI	790 (8.7)	134 (10.3)	783 (6.6)	
DNR/OkInt	329 (3.6)	143 (11)	209 (1.8)	
CMO	1086 (12.0)	913 (70.1)	223 (1.9)	
Discharged home	2308 (25.4)			
Discharged to hospice				
Hospice-home	137 (1.5)			
Hospice-medical facility	261 (2.9)			
Patients died	1259 (13.9)			
COVID status				<0.0001
COVID +	581 (6.4)	175 (13.9)	406 (5.2)	
Prior COVID +	33 (0.4)	5 (0.4)	28 (0.4)	
Not COVID +	8469 (93.2)	1079 (85.7)	7390 (95.5)	
LOS				0.1
Median (IQR)	8 (4, 15)	8 (3, 17)	6 (3, 11)	
Mean (SD)	12.7 (16.1)	12.7 (15.8)	9.8 (14)	
[min, max]	[1, 384]	[1, 127]	[1, 384]	
ICU LOS				<0.0001
Median (IQR)	2.7 (1.3, 6.1)	4 (1.4, 9.3)	2.1 (1, 4.8)	
Mean (SD)	5.5 (8.5)	7.3 (9.8)	4.3 (7.2)	
[min, max]	[0, 170.3]	[0, 127.9]	[0, 170.3]	
SOFA for first 24 hours				<0.0001
Median (IQR)	2 (1, 4)	4 (3, 4)	2 (1, 4)	
Mean (SD)	2.4 (1.8)	3.8 (1.8)	2.2 (1.7)	
[min, max]	[0, 12]	[0, 12]	[0, 11]	

Using column percentages^**^.

CMO, Comfort Measures Only; DNI, Do Not Intubate; DNR, Do Not Resuscitate; ICU, intensive care unit; IQR, interquartile range; LOS, length of stay; max, maximum; min, minimum; OkInt, ok to intubate; SD, standard deviation; SOFA, sequential organ failure assessment.

Fourteen percent of those who died were COVID-19 positive as compared with those who were discharged alive (5.2%) (*p* < 0.0001). In 2270 patients (25%), the race and ethnicity were “Unknown.” There was a significant difference in the overall racial and ethnic breakdown of the decedents as compared with those discharged alive (*p* < 0.0001), with a greater proportion of individuals who were of “Unknown” race dying (22.7%) as compared with those who were alive (11.5%).

Similarly, a significant proportion of the patients who survived identified as non-Hispanic (82.7%) or White (64.8%) (*p* < 0.0001).

In addition, a subset of 258 patient charts were manually reviewed to derive an exploratory estimate of how often “Unknown” race can be determined from the EHR. Only 14% of patients had race data that could be identified based on descriptors available in the chart.

### Codes status and timing among decedents

The majority of those who survived were Full code (89.8%). Of the decedents, 21.3% had a limitation of resuscitation (DNR/Ok to intubate or DNR/DNI) but were not transitioned entirely to comfort measures, and 70.1% had a CMO order (*p* < 0.0001). Table 2 shows that the code status varied by race, with a higher percentage of White patients designated as CMO, 74% compared with 65.7% of Black patients, whereas for DNR/DNI or DNR/Ok to intubate 27.8% were Black patients, followed by 23.8% of “other” race and 22.7% of “Unknown” race.

**Table 2.** Interventions at Time of Death by Race/Ethnicity

Intervention present at time of death	All patients (***n*** = 1259), ***n*** (%)	American Indian/Alaska Native and Native Hawaiian/Other Pacific Islander (***n*** = 9)	Asian (***n*** = 39)	Black (***n*** = 166)	White (***n*** = 717)	Other (***n*** = 42)	Unknown (***n*** = 286)	***p***
Code status								0.006
CMO	880 (69.9)	5 (55.6)	24 (61.5)	109 (65.7)	531 (74.1)	24 (57.1)	187 (65.4)	
DNR/DNI	130 (10.3)	2 (22.2)	6 (15.4)	20 (12.1)	73 (10.2)	3 (7.1)	26 (9.1)	
DNR/OkInt	143 (11.4)	0 (0)	4 (10.3)	26 (15.7)	67 (9.3)	7 (16.7)	39 (13.6)	
Full code	106 (8.4)	2 (22.2)	5 (12.8)	11 (6.6)	46 (6.4)	8 (19.1)	34 (11.9)	
Intubation	557 (44.2)	3 (33.3)	15 (38.5)	68 (41)	312 (43.5)	14 (33.3)	145 (50.7)	0.12
Vasopressors	653 (51.9)	7 (77.8)	17 (43.6)	88 (53)	364 (50.8)	19 (45.2)	158 (55.2)	0.30
Tube feeding orders	98 (7.8)	0 (0)	1 (2.6)	9 (5.4)	50 (7)	4 (9.5)	34 (11.9)	0.07
Dialysis	207 (16.4)	1 (11.1)	1 (2.6)	39 (23.5)	106 (14.8)	3 (7.1)	57 (19.9)	0.002
Restraint orders	278 (22.1)	2 (22.2)	7 (18)	36 (21.7)	168 (23.4)	7 (16.7)	58 (20.3)	0.78
Palliative care consult	355 (28.2)	1 (11.1)	11 (28.2)	53 (31.9)	218 (30.4)	15 (35.7)	57 (19.9)	0.01
Ethics consult	32 (2.5)	0 (0)	0 (0)	5 (3)	19 (2.7)	1 (2.4)	7 (2.5)	0.96

Using column percentages^**^.

The median time between DNR decision and death was 11.5 hours (IQR 5.4, 19.5) for patients of “other” race, 10.3 hours (IQR 4.6, 30.5) for Black patients, and 7.9 hours (IQR 4.1, 18.1) for White patients, suggesting an earlier decision by patients who identified as Black compared with White (Supplementary Table S2). Median time to death from a DNR decision was significantly different between Black and White patients (*p* < 0.01).

**FIG. 2.**
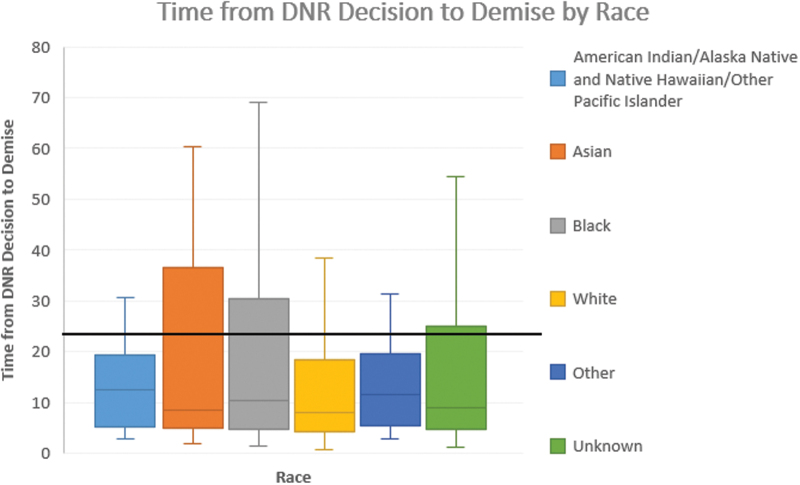
Boxplot of time from DNR decision until death by race.

Figure 2 illustrates the wide variation among all race groups, with Black patients having the widest variation overall (*p* = 0.07) (Supplementary Fig. S1). It is difficult to speculate whether these differences are due to differences in treatment preferences or other factors such as physician practice in offering comfort-focused care. It has been shown that physicians frequently do not offer comfort-focused care as an option, and it has yet to be explored how patient race and physician implicit bias influences these discussions.^13^

### Markers of intensity of care as well as communication

For those who died, the type of care received was categorized by markers of intensity of care and markers of communication around goals of care. Overall, at the time of death, 44.2% of patients were intubated, 52% of patients were receiving vasopressors, 7.8% of patients had tube feeding orders, 16.4% of patients were on dialysis, and 22% of patients had restraint orders (Table 2).

Patients with race designated as “Unknown” were also more likely to be intubated (50.7%), receiving tube feeds (11.9%), and receiving dialysis (20%). There were overall significant differences in consulting Palliative care as well among the different races, with patients of “Other” race receiving the most consults (35.7%) (*p* < 0.01).

There was discordance between White and all other race patients with respect to rate of Palliative care consults (30% vs. 25%, *p* < 0.04) (Supplementary Table S3). Overall, there were very few Ethics consults, and no significant difference between race categories was found.

Table 3 shows interventions present at the time of death adjusted for age and SOFA score. Patients of “Unknown” race appeared significantly more likely to die intubated, and on vasopressors, as well as with a “Comfort measures only” (CMO) order in place (*p* < 0.001). Patients who self-identified as “Other” were significantly less likely to have a CMO order (*p* < 0.011).

**Table 3.** Interventions Present at Time of Death, Adjusted for Age and Sequential Organ Failure Assessment Score, Odds Ratio (95% Confidence Interval)

	American Indian/Alaska Native and Native Hawaiian/Other Pacific Islander	Asian	Black	White	Other	Unknown	Age	SOFA score
CMO	0.644 (0.268–1.545)	0.84 (0.569–1.239)	0.889 (0.722–1.095)	Ref.	*0.588 (0.39–0.887)*	*1.588 (1.325–1.905)*	1.042 (1.037–1.047)	1.626 (1.562–1.692)
Intubation	0.686 (0.237–1.985)	0.686 (0.398–1.184)	1.143 (0.897–1.456)	Ref.	0.634 (0.387–1.038)	*2.106 (1.72–2.578)*	1.011 (1.006–1.016)	1.011 (1.609–1.763)
Vaso-pressors	1.539 (0.691–3.428)	0.84 (0.512–1.38)	0.998 (0.776–1.282)	Ref.	0.673 (0.414–1.096)	*1.866 (1.516–2.298)*	1.023 (1.018–1.029)	1.771 (1.689–1.857)
Tube feed-ing orders	0.248 (0.015–4.151)	1.277 (0.693–2.354)	1.203 (0.855–1.695)	Ref.	0.96 (0.521–1.768)	1.346 (0.968–1.872)	1.007 (0.999–1.014)	1.274 (1.199–1.354)
Dialysis	8.880 (1.451–54.329)	0.846 (0.05–14.374)	*3.713 (1.474–9.353)*	Ref.	1.504 (0.267–8.477)	*3.568 (1.365–9.328)*	1.015 (0.991–1.039)	*1.7 (1.436–2.013)*
Restraint orders	1.669 (0.223–12.489)	1.076 (0.332–3.490)	1.550 (0.886–2.711)	Ref.	1.771 (0.790–3.970)	*2.709 (1.649–4.451)*	*1.026 (1.012–1.039)*	*1.312 (1.178–1.461)*
Palliative care consult	1.315 (0.400–4.323)	*2.180 (1.382–3.438)*	*1.741 (1.318–2.229)*	Ref.	0.964 (0.570–1.631)	0.502 (0.314–0.804)	*1.012 (1.004–1.019)*	*1.223 (1.151–1.300)*
Ethics consult	1.672 (0.095–29.313)	1.348 (0.256–7.097)	0.798 (0.257–2.476)	Ref.	0.862 (0.164–4.532)	0.633 (0.171–2.349)	1.023 (0.998–1.050)	*1.588 (1.337–1.886)*

Italics are significant results.

There was a trend toward Black patients being less likely to die with Comfort measures, but this was not statistically significant. Use of dialysis at the end of life varied by self-identified race. Specifically, Black and Unknown patients were more likely to receive renal replacement therapy, even after adjustment for age and severity of illness (24% and 20%, *p* = 0.003).

### Subgroup analysis

#### Pre– and post–COVID-19 pandemic surge

Supplementary Table S4 shows a comparison of patients admitted before and after the first reported COVID-19 patient in our ICUs in March 2020. The proportion of patients who were of Black, “Unobtainable” ethnic group, or “Unknown” race increased compared with before the pandemic. The percentage of Black patients increased from 12.8% to 15.4%.

This was reversed for White patients, where pre-COVID pandemic, 65.5% of admitted patients were White compared with 62.3% after the start of the pandemic in March 2020 (*p* = 0.003). The overall mortality increased after the pandemic began, from 13.4% to 14.2%.

Fewer patients were made DNR/DNI after the pandemic (9.5% compared with 8.1%), but the number who were DNR/Ok to intubate increased from 3% to 4% (*p* = 0.016). The median ICU LOS also increased after the pandemic (from 2.3 (IQR 1.1, 4.9) days to 3 (IQR 1.4, 7) days (*p* < 0.0001).

## Discussion

In this descriptive study of end-of-life care in critically ill patients at a single institution, we found a significant portion of patients not having a designated race or ethnicity according to the OMB categories.^14^ There are multiple possible reasons why these patients might be missing race data: they could have been too sick to go through the standard registration process, they could be a group who has less prior health care contact for reasons related to their race or non-race related reasons, or they could be patients who declined to respond to race related questions during prior health care contact.

Although we do not know whether the “unknown” race patients were non-White, their outcomes and trajectories matched non-White patients. Regardless, these patients differed in the aggressiveness of care that they received at the end of life. More White patients were designated as CMO at the time of death (comfort-focused care), as compared with “Unknown” race and Black patients.

One possibility in these observed differences may be that White patients wish to limit care more than other patients. Another hypothesis is that there are increased communication challenges that may reflect a breakdown of trust between the care team and non-White patients or their families.^15^ Black patients had a longer time (even if by a few hours) between DNR decision and death. One could hypothesize multiple reasons for this gap, including different barriers to accessing the hospital (such as access to transportation, ability to miss work, etc.) or different cultural or religious processes before death.

The implication of having a large population of patients without an identified race could mean that these patients are either undocumented (or without prior identification), or do not identify as any of the race options offered in structured EHRs. Previously, Sholle et al. have pointed out limitations to data quality when EHR is used alone to identify race.^16^

In their study of 16,000 plus patients, they found that a quarter of patients with missing race in EHR were found to be Black or Hispanic, older, and with more comorbidities. In our data review, a subset of 258 patient charts were reviewed by hand, and of those only 38 patients (14%) had race data that could be identified based on descriptors available in the chart.

Of those, 15 (39% of those able to be identified) were White, 9 (23.6%) were Black, and 7 were Hispanic (18.4%). Supplementing structured race data with open-ended choices or spoken language derived race and ethnicity data may help in assessing demographic makeup and outcomes of patients who are not identified. This would allow researchers and policy makers to draw more accurate conclusions about differences in racial outcomes at the end of life.

As it is likely that the patients in the “Unknown” group are not missing completely at random, we are unable to draw causal conclusions about the explanation for differing intensity of care by non-missing race categories. However, that we identified any differences points to heterogeneity in how end-of-life care is delivered, which does appear to vary based on patient demographics rather than severity of illness.

This raises the question of whether this heterogeneity is due to variations in goals of care or due to differences in physician behavior related to perceptions that may be influenced by systemic bias. For example, the question arises as to whether care teams might ask questions related to code status sooner or recommend a DNR/DNI code status sooner if they perceive a patient as sicker, which may be influenced by patient race.

In our study, the “Unknown” race group had significantly higher rates of death, were sicker on admission, and had more aggressive measures of care at the end of life compared with other patients, as well as received significantly more comfort measures at death. The absence of any self-identified or designated race may serve as a signal that this population could be less able to provide care preferences, or their prior goals and values may be difficult to determine, leading to more aggressive care.

There were differences in consulting Palliative care. The addition of input from Palliative care teams has been shown to improve the quality of death and support for the dying patients and their families.^17^ However, recently, literature points toward inequity among cancer patients of underserved racial groups being offered such referrals.^18^

Also, prior literature has shown that conflicts and other cognitively based concerns requiring mediation trigger greater clinical Ethics consultations.^19^ Black patients received less comfort care at death; however, this was not significant, and received more dialysis at death after adjustment for age and severity of illness.

These data support a growing body of literature suggesting that there are differences in the end-of-life care provided to patients of diverse racial and ethnic backgrounds in the ICU. These may arise due to differences in patient and family preferences, physician practice impacting communication, and extent of interaction with the medical system before ICU admission. Although comfort-focused care may often seem most appropriate in a patient for whom a physician understands the prognosis is very poor, this may not always be the best choice for a patient or their family.^20^

Given the range in cultural beliefs surrounding death, as long as care in the ICU at the time of death is concordant with the goals, preferences, and beliefs of the patient, this should be considered a good death.^21^ Discordance in goals of care occurs because of lack of documented preferences,^22^ and poor communication,^23^ including intensivists being indirect about prognosis,^24^ and often thinking they have offered care focused on comfort when they have not.^25^

In addition to the general challenges that exist with end-of-life care in the ICU, there are also disparities in end-of-life care in the United States more generally.^26^ Literature shows that underserved racial groups receive more aggressive and potentially non-beneficial medical care at the end of life, and less information about a diagnosis, prognosis, and treatments.^5^

Our study leads to further generation of hypotheses, including whether such differences are due to racial bias among care providers, and whether preferences differ along racial lines due to cultural differences in end-of-life care goals. The COVID 19 pandemic led to worsening of outcomes among patients; however, the differences in racial outcomes persisted.

Our study had several limitations. These data were retrospective and extracted from the electronic medical record. Individual chart review to better assess the quantity or quality of family discussions, or the thought process behind the decisions being made was not performed.

This can lead to bias when attempting to understand the data. In addition, although the sample size was fairly large, a significant proportion of race and ethnicity data (12% and 13%) was “Unobtainable or Unknown,” and an additional 5% of patients identified as a category “Other” than the provided options during the process of hospital registration.

Further, using self-identified or designated race collected at the time of hospital registration to categorize individuals is a limited window into the multifaceted ways in which a patient's appearance, personal beliefs about their race, and cultural background may affect their interactions with the health care system.

One striking finding in our data is that almost a quarter of the dying patients did not have an identified race or ethnicity. Race and ethnicity data are important to support the evaluation of personalized medical initiatives and to assess for the presence of health care inequities.

Another novel finding was the significant decrease in rate of DNR/DNI patients after the pandemic began, potentially as a result of visitor restrictions limiting communication with family members on these end-of-life decisions. Based on these findings, further work needs to be done to implement changes in ICU care of all racial groups and to pro-actively consider changes such as triggers for Palliative care medicine consults for dying patients of racial minorities to encourage ICU teams to consider these practices.

Social work liaisons should also be involved earlier for communication with patients whose racial and ethnic groups are unknown or those who are not represented equally. Finally, a greater emphasis from ICU team leaders and staff to improve these differences uncovered in this study. Engagement with patient group liaisons and stakeholders can also help in communication and trust building between physicians and family members.^27^

## Conclusion

These data describe several differences in characteristics of care before death in an ICU by race, as well as the potential areas of difference between patients identifying with different race categories. A major hurdle in improving care at the end of life is a poor understanding of cultural differences related to care preferences. If the differences we found exist because of differences in how the care team works with the patients and families, then this is a major gap in end-of-life care.

Further studies done prospectively and with a qualitative patient-focused view will be crucial in evaluating these differences in the care of vulnerable patients at the end of life. It is crucial to determine whether these differences result from disparities, and whether these disparities exist due to systemic racial biases in the ICU care received by patients of differing race and ethnicities.

## Supplementary Material

Supplemental data

Supplemental data

Supplemental data

Supplemental data

Supplemental data

## Abbreviations Used

CMO: Comfort Measures Only
DNI: Do Not Intubate
DNR: Do Not Resuscitate
EHRs: electronic health records
ICU: intensive care unit
IQR: interquartile range
LOS: length of stay
Max: maximum
Min: minimum
OkInt: ok to intubate
OMB: Office of Management and Budget
SD: standard deviation
SOFA: sequential organ failure assessment
