# Supplementary material for: Examining the Role of Race in End-of-Life Care in the Intensive Care Unit: A Single-Center Observational Study
Source: Palliat Med Rep. 2023 Sep 11;4(1):264–73. doi: 10.1089/pmr.2023.0037 (PMC10507941; doi:10.1089/pmr.2023.0037)
Supplement: Supplemental data [file Suppl_TableS1.docx]

**Supp Table 1 a. Discharge disposition by race**

**
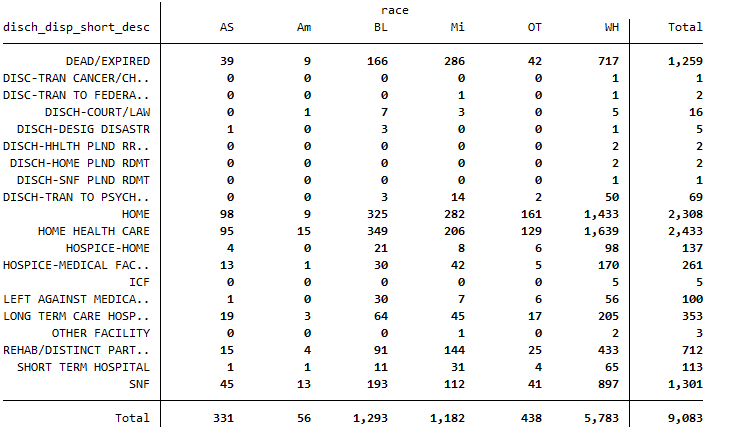
**

**Supp Table 1b. Detailed Discharge Disposition**

| **Discharge Disposition** | **Number** | **Percent (n=9083)** |
| --- | --- | --- |
| Dead | 1259 | 13.9 |
| Hospice |  |  |
| Home | 137 | 1.5 |
| Medical Facility | 261 | 2.9 |
| Home | 2308 | 25.4 |
| Home Health Care | 2433 | 26.8 |
| Long Term Care |  |  |
| Hospital | 353 | 3.9 |
| Other Facility | 3 | 0.03 |
| Rehab/Distinct Part Hospital | 712 | 7.8 |
| Short Term Care |  |  |
| Hospital | 113 | 1.2 |
| SNF (Short term nursing facility) | 1301 | 14.3 |
| ICF (Intermediate care facility) | 5 | 0.06 |
| Other |  |  |
| Transferred to Cancer/Children’s Hospital | 1 | 0.01 |
| Transferred to Federal HC | 2 | 0.02 |
| Transferred to Psych Hospital | 69 | 0.8 |
| Court/Law | 16 | 0.2 |
| DESIG DISASTR | 5 | 0.06 |
| HHLTH (Home health) | 2 | 0.02 |
| HOME | 2 | 0.02 |
| SNF (Short term nursing facility) | 1 | 0.01 |
| Left Against Medical Advice | 100 | 1.1 |
