# Supplementary material for: Examining the Role of Race in End-of-Life Care in the Intensive Care Unit: A Single-Center Observational Study
Source: Palliat Med Rep. 2023 Sep 11;4(1):264–73. doi: 10.1089/pmr.2023.0037 (PMC10507941; doi:10.1089/pmr.2023.0037)
Supplement: Supplemental data [file Suppl_TableS2.docx]

**Supp Table 2. Time between DNR decision and demise by race (only among patients who died and who were not full code (n=1153)**

| Time between DNR decision and demise (in hours) | **American Indian/Alaska Native and Native Hawaiian/Other Pacific Islander (n=7)** | **Asian (n=34)** | **Black (n=155)** | **White (n=671)** | **Other (n=34)** | **Unknown (n=252)** | **Total Group (n=1153)**  **(p=0.071)** |
| --- | --- | --- | --- | --- | --- | --- | --- |
| Mean (SD)  Median (IQR)  [min, max] | 13.5 (10.7)  12.4 (5.2, 25.5)  [2.8, 30.5] | 32.2 (45.4)  8.5 (4.6, 39)  [2, 167.8] | 40 (79.6)  10.3 (4.6, 30.5)  [1.5, 488.7] | 25.8 (106.7)  7.9 (4.1, 18.4)  [0.7, 2396.4] | 17.1 (19.3)  11.5 (5.4, 19.5)  [2.8, 92.9] | 23.7 (39.1)  9 (4.7, 25)  [1.2, 332.5] | 27.1 (88.9)  8.5 (4.4, 21.9)  [0.7, 2396.4] |
