# Supplementary figures and images for: Examining the Role of Race in End-of-Life Care in the Intensive Care Unit: A Single-Center Observational Study
Source: Palliat Med Rep. 2023 Sep 11;4(1):264–73. doi: 10.1089/pmr.2023.0037 (PMC10507941; doi:10.1089/pmr.2023.0037)

**Supplemental Figure 1. Time from DNR to demise between Blacks and Whites only**


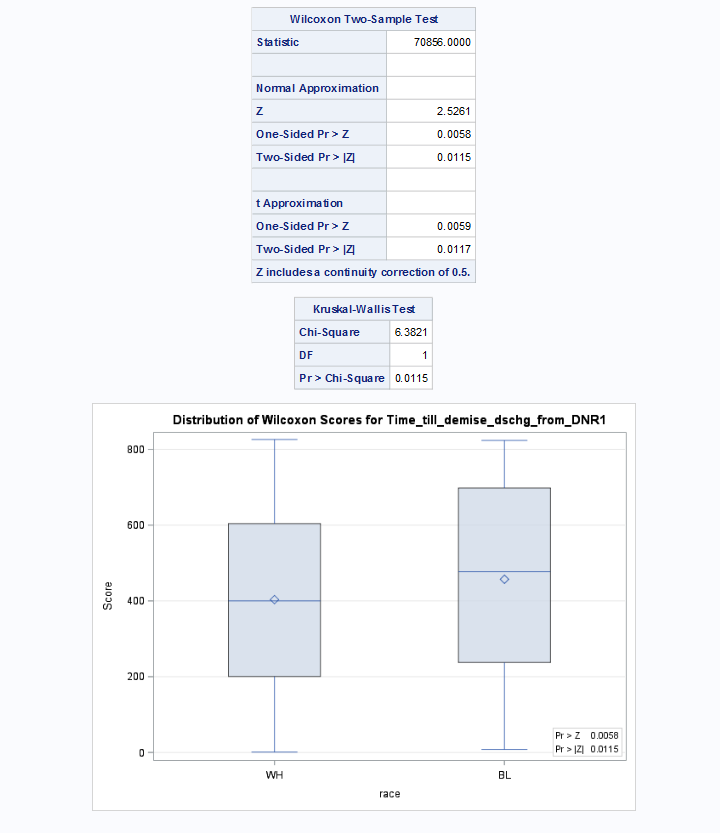

Supplement: Supplemental data [file Suppl_FigureS1.docx]
