# Supplementary material for: Examining the Role of Race in End-of-Life Care in the Intensive Care Unit: A Single-Center Observational Study
Source: Palliat Med Rep. 2023 Sep 11;4(1):264–73. doi: 10.1089/pmr.2023.0037 (PMC10507941; doi:10.1089/pmr.2023.0037)
Supplement: Supplemental data [file Suppl_TableS3.docx]

**Supplemental Table 3. Interventions at death comparing White patients with all other races**

| **Intervention Present at time of Death** | **All Patients (n=1259), No. (%)** | **White**  **(n=717)** | **Other Races (n=542)** | **p-value** |
| --- | --- | --- | --- | --- |
| Code Status,  CMO  DNAR/DNI  DNAR/ Ok Int  Full Code | 880 (69.9)  130 (10.3)  143 (11.4)  106 (8.4) | 531 (74.1)  73 (10.2)  67 (9.3)  46 (6.4) | 349 (64.4)  57 (10.5)  60 (11.1)  76 (14) | 0.0004 |
| Intubation | 557 (44.2) | 312 (43.5) | 245 (45.2) | 0.55 |
| Vasopressors | 653 (51.9) | 364 (50.8) | 289 (53.3) | 0.36 |
| Tube Feeding orders | 98 (7.8) | 50 (7) | 48 (8.9) | 0.21 |
| Dialysis | 207 (16.4) | 106 (14.8) | 101 (18.6) | 0.06 |
| Restraint orders | 278 (22.1) | 168 (23.4) | 110 (20.3) | 0.18 |
| Palliative care consult | 355 (28.2) | 218 (30.4) | 137 (25.3) | 0.04 |
| Ethics consult | 32 (2.5) | 19 (2.7) | 13 (2.4) | 0.77 |
