# Supplementary material for: Examining the Role of Race in End-of-Life Care in the Intensive Care Unit: A Single-Center Observational Study
Source: Palliat Med Rep. 2023 Sep 11;4(1):264–73. doi: 10.1089/pmr.2023.0037 (PMC10507941; doi:10.1089/pmr.2023.0037)
Supplement: Supplemental data [file Suppl_TableS4.docx]

**Supp Table 4. Pre and post pandemic surge (col%)**

| **Variable** | **Pre-COVID (n=3891)** | **Post-COVID (after March surge 2020) (n=5192)** | **p-value** |
| --- | --- | --- | --- |
| Code status, No. (%)  DNR/DNI  DNR/OkInt  CMO  Full Code | 369 (9.5)  120 (3.1)  466 (12)  2936 (75.5) | 421 (8.1)  209 (4)  620 (11.9)  3942 (75.9) | 0.015 |
| Gender, No. (%)  Male | 2148 (55.2) | 2944 (56.7) | 0.15 |
| Ethnicity, No. (%)  Non-Hispanic/Latino  Unobtainable  Hispanic/Latino | 3191 (82)  448 (11.5)  252 (6.5) | 4213 (81.1)  640 (12.3)  339 (6.5) | 0.48 |
| Race, No. (%)  American Indian/Alaska Native and Native Hawaiian/Other Pacific Islander  Asian  Black  Other  Unknown  White | 30 (0.8)  137 (3.5)  496 (12.8)  189 (4.9)  492 (12.6)  2547 (65.5) | 26 (0.5)  194 (3.7)  797 (15.4)  249 (4.8)  690 (13.3)  3236 (62.3) | 0.003 |
| Age, Mean (SD) | 64.6 (17.1) | 64.2 (16.5) | 0.37 |
| Discharged Home, No. (%) | 974 (25) | 1334 (25.7) | 0.47 |
| Discharged to Hospice, No. (%) | 179 (4.6) | 219 (4.2) | 0.37 |
| Patients died, No. (%) | 523 (13.4) | 736 (14.2) | 0.31 |
| LOS  Median (IQR) | 8 (4, 16) | 8 (4, 15) | 0.009 |
| ICU LOS  Median (IQR) | 2.3 (1.1, 4.9) | 3 (1.4, 7) | <0.0001 |
| SOFA Scores  Median (IQR) | 2 (1, 4) | 2 (1, 4) | <0.0001 |
